# Examination Techniques of the First Cranial Nerve: What Neurosurgical Residents Should Know

**DOI:** 10.21315/mjms2020.27.5.12

**Published:** 2020-10-27

**Authors:** Leonard Leong Sang Xian, Vasu Nallaluthan, Yong De Jun, Ooi Lin-Wei, Sanihah Abdul Halim, Chee Yong Chuan, Zamzuri Idris, Abdul Rahman Izaini Ghani, Jafri Malin Abdullah

**Affiliations:** 1 Department of Neurosciences, School of Medical Sciences, Universiti Sains Malaysia, Kubang Kerian, Kelantan, Malaysia; 2 Hospital Universiti Sains Malaysia, Universiti Sains Malaysia, Kubang Kerian, Kelantan, Malaysia; 3 Department of Neurosurgery, Hospital Sungai Buloh, Selangor, Malaysia; 4 Department of Neurosurgery, Hospital Umum Sarawak, Kuching, Sarawak, Malaysia; 5 Neurology Unit, Department of Internal Medicine, School of Medical Sciences, Universiti Sains Malaysia, Kubang Kerian, Kelantan, Malaysia; 6 Brain and Behaviour Cluster, School of Medical Sciences, Universiti Sains Malaysia, Kubang Kerian, Kelantan, Malaysia

**Keywords:** olfactory nerve examination, smell detection threshold, smell discrimination, smell identification

## Abstract

Olfactory or smell dysfunction is often overlooked by clinicians despite being prevalent in the population. To date in Malaysia, there is no standard and reliable test to examine the function of olfaction. Tests used at developed countries such as the Sniffin’ Sticks Test (SST), the Connecticut Chemosensory Clinical Research Center (CCCRC) test, the University of Pennsylvania Smell Identification Test (UPSIT) and the Brief Smell Identification Test (B-SIT) are not readily available in this region and may be costly to procure. The first cranial nerve can be tested using commonly available materials to assess: i) the function of odour detection; ii) the odour discrimination; and iii) the odour identification. An abnormal odour detection threshold test generally indicates a peripheral olfactory problem while the odour discrimination and identification test attribute the problem to the cerebral cortex. An olfactory complaint should not be taken lightly and a proper olfactory function examination is important: i) to determine the legitimacy of a patient’s complaint; ii) to monitor the progress of patient’s olfactory function; iii) to establish insurance payout for disability; and iv) to characterise the specific nature of the problem.

A video has been produced to demonstrate the examination techniques explained in this article.

## Introduction

Smell dysfunction is a common disorder, yet it is often overlooked by the clinicians. An impaired smell can negatively impact a person’s life as it changes the taste of food and drinks. Furthermore, it may pose a potential health hazard since the person will not be able to detect leaking gas, smoke or spoiled/contaminated food. This condition is also detrimental to the employees that depend on their ability to smell in their profession and safety such as firefighters, plumbers, chefs, perfume sellers and workers in chemical or gas industries.

Currently, there is no standardised olfactory nerve examination in Malaysia. The common smell tests used at developed countries include the Sniffin’ Sticks Test (SST) (1–2), the Connecticut Chemosensory Clinical Research Center (CCCRC) test (1), the University of Pennsylvania Smell Identification Test (UPSIT) (1–2) and the Brief Smell Identification Test (B-SIT) (2), which are not readily available in this region and may not be economically viable to use in some centres. Therefore, it is essential to establish a standardised and reproducible examination technique of the first cranial nerve that can be performed by the Malaysian doctors, nurses, and medical students using the readily available materials.

A cranial nerve I (CN I) examination assesses the ability of a person: i) to detect; ii) to discriminate; and iii) to identify an odour (1, 3).

## Odour Detection Threshold Test

Odour detection threshold can be tested using the alcohol sniff test (AST), which was first described by Davidson and Murphy (4) in 1997. There were 100 adult participants in the original study consisting of 64 patients with peripheral nasal diseases and 36 healthy individuals as controls (the significant difference between the subjects and controls, *P* < 0.003) (4). A year later, Davidson et al. (5) replicated the AST on 46 normal children within the age of 6–15 years old (test-retest correlation *P* < 0.0001). This test categorises the patients into normosmia, hyposmia and anosmia groups.

### Materials

- One standard 70% isopropyl alcohol pad
- One 30 cm metric ruler

### Technique

**Figure 1:**
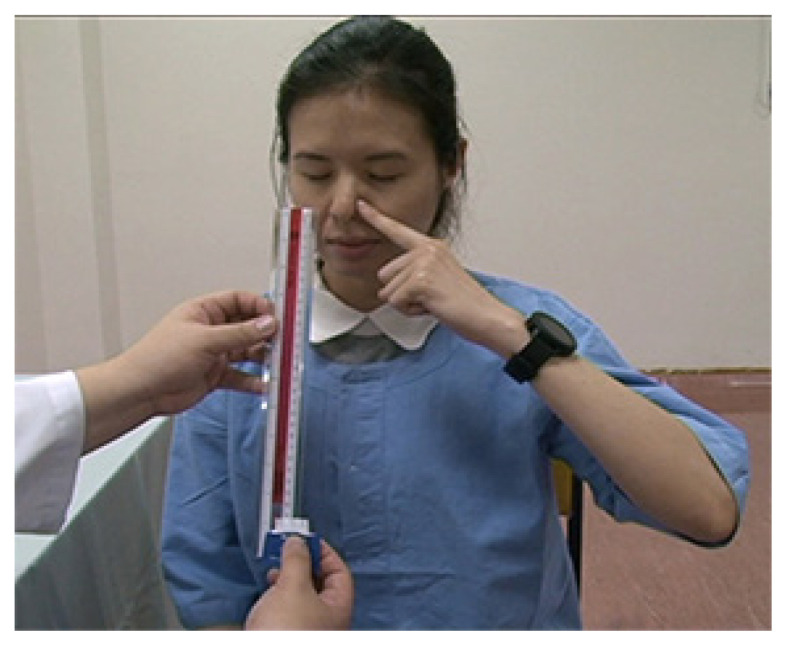
Patient is instructed to close her eyes and occlude one of her nostrils using her finger. Alcohol pad is moved up 1 cm with each exhalation until she indicates that she has detected the smell

- The alcohol pad wrapper is torn to expose approximately 5 mm of the pad
- The patient is allowed to familiarise with the alcohol scent by placing the pad beneath their nostrils
- A 30 cm ruler is placed downward with the 0 cm mark at the patient’s nose
- The patient is instructed to close their eyes and mouth and obstruct one of the nostrils using a finger. The nostril that may be abnormal should be examined first
- Starting from the 30 cm mark, the alcohol pad is introduced and moved 1 cm closer to the nose with each exhalation until the alcohol scent is perceived (Figure 1)
- The distance between the alcohol pad and the nose is recorded
- The test is repeated with the other nostril after a recovery period of 45 s

#### The distance from nostril to the alcohol pad (4–5)

##### Adult

- Anosmia: 0 cm–7 cm
- Hyposmia: > 7 cm–12 cm
- Normosmia: > 12 cm

##### Children (6–15 years old)

- Anosmia: 0 cm–5 cm
- Hyposmia: > 5 cm–12 cm
- Normosmia: > 12 cm

## Odour Discrimination and Identification Test

Odour discrimination test (6) is used to determine if a patient can distinguish between odorous and odourless substances. The odour identification test requires the patient to identify the substance. These two tests are performed simultaneously using five different types of substances (Table 1). Saiki (6) published this method in 2003 that was developed based on T & T Olfactometer (a standardised olfactometer used in Japan). The data were derived from a large sample of healthy men and women in the age between 18 and 25 years old (7).

### Materials

- Sets of five smelling strips (one set for each substance)
  - numbered 1–5
  - 14 cm long × 0.7 cm wide (cut from a 180 gsm–200 gsm white paper)
- One binder clip to hold the smelling strips
- Five bottles of odour solutions containing the substances listed in Table 1 (the scents that are used are those commonly found in the local population)
- One bottle containing odour-free liquid paraffin as control
- One holding stand for smelling strips that have been sniffed by the patient
- One board to separate the examiner and the patient to avoid revealing the correct answers

### Technique

- The hands of the examiner and the examinee are checked if they carry any odour. If any odour is detected, they are asked to wash their hands with an odour-free soap
- Five colourless odour solutions and one odourless control solution are used
- The two-out-of-five test (choosing two smelling strips out of five) is implemented to reduce the probability of the patients guessing the answers correctly
- A clip is used to bind the five smelling strips together
- Two random smelling strips are dipped into the odour solution with approximately 1 cm deep. The tip is wiped against the inside wall of the bottle after dipping to prevent dripping
- The tips of the other three strips are immersed into liquid paraffin as control
- The set of the smelling strips is passed to the patient
- The patient is instructed to sniff the odour by placing the tip of the smelling strip 1 cm–2 cm from their nose for not more than five seconds for each strip
- The smelling strips that have been sniffed will be placed into the holding stand
- After sniffing the odour of all five smelling strips, the patient is asked to provide the number of the two smelling strips with odour and to identify the smell on a piece of paper provided
- If the patient is unable to discern the odour, the patient will be given a second chance to sniff the smelling strips
- The used smelling strips are discarded into a covered wastebasket after each test to prevent residual odour in the examination room
- The test is repeated for the rest of the odour solutions in the order listed in Table 1

### Interpretation

- A patient who answers correctly for all five odour solutions is considered normal.
- If a patient can answer four out of five odour solutions, the test is repeated twice with the odour solution for the incorrect answer. If the patient can give correct answers for the two repeated tests, the patient is considered to have successfully passed the olfactory test.

## Discussion

An abnormal odour detection threshold test generally indicates a peripheral olfactory problem. Contrarily, odour discrimination and identification test are attributed to a problem in the cerebral cortex (1, 3).

The odour detection threshold test should be performed prior to the odour discrimination and identification tests, as a defect in smell detection may not yield a reliable result for the subsequent tests. Trigeminal thresholds for alcohols are two or more orders of magnitude higher than the odour detection threshold (5). Therefore, alcohol scent can only be perceived by an anosmic if it is at extreme proximity to the nose (5).

In the odour discrimination and identification test, smelling strips are used instead of directly advancing the bottles to the patient’s nostril as hand odour and odour attached to the outside of the bottle will contaminate the sensing of the smell (7). The odours are tested from weak to a strong smell. This is because a stronger odour will decrease the sensitivity of smelling the subsequent weaker odour (7).

Landis et al. (1) reported that the common aetiologies of smell dysfunction are sinunasal problems, post-trauma, post-upper respiratory tract infections and neurodegenerative diseases. Less commonly, it can also be caused by endocrine disorders, epilepsy, drug/toxin and of congenital origin.

Smell deficits usually precede the symptoms of neurodegenerative diseases such as dementia, Alzheimer’s disease (AD), Huntington’s disease, motor neuron disease, and Parkinson’s disease (1, 2, 8, 9). Therefore, an olfactory dysfunction should raise the suspicion of the previously mentioned diseases. An asymmetrical odour detection threshold between the left and right nostrils of more than 5 cm can also be used as an early predictor of AD (9).

## Conclusion

In conclusion, an olfactory complaint should not be taken lightly and a proper olfactory function examination is important: i) to ascertain the legitimacy of a patient’s complaint; ii) to follow up the progress of patient’s olfactory function; iii) to establish insurance payout for disability; and iv) to characterise the specific nature of the problem (3, 8). The methods described above may provide an objective assessment of the first cranial nerve.

A video ( https://youtu.be/LYMiTYr5amU ) has been produced to demonstrate the examination techniques explained in this article.

## Figures and Tables

**Figure 2:**
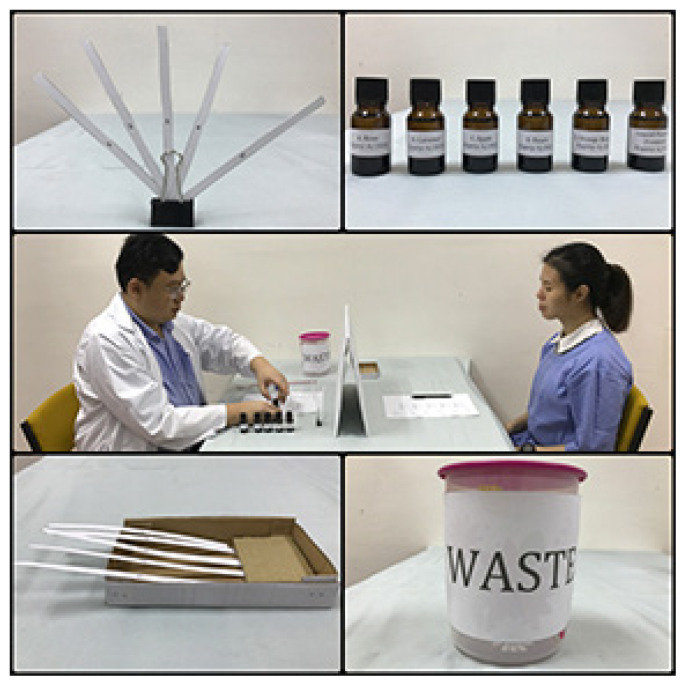
Clockwise from top left: Five numbered smelling strips attached to a binder clip, bottles containing five different odour solutions and liquid paraffin as control, the layout of odour discrimination and identification test, a covered wastebasket and a holding stand for used smelling strips

**Table 1:** List of substances being tested and examples of scents containing the substance

No	Name of substance	Examples of scents containing the substance
1	β-Phenylethyl alcohol	Rose, carnation
2	Methyl cyclopentenolone	Caramel, maple syrup, coffee, wine, paprika, salmon
3	Isovaleric acid	Cheese, soy milk, apple juice
4	ϒ-Undecalactone	Peach, apricot, strawberry
5	Skatole (3-methyl indole)	Orange blossom, jasmine
